# Survival modeling and assessing prognostic factors of colorectal cancer in Ethiopia: Addressing violation of statistical assumptions

**DOI:** 10.1371/journal.pone.0361233

**Published:** 2026-10-26

**Authors:** Tuem Gebre Abraha, Mulu Reda Terefe, Zeytu Gashaw Asfaw

**Affiliations:** 1 Department of Statistics, College of natural and computational sciences, Adigrat University, Adigrat, Ethiopia; 2 Department of Statistics, Hawassa University, Hawassa, Ethiopia; 3 Department of Epidemiology and Biostatistics, School of Public Health, Addis Ababa University, Addis Ababa, Ethiopia; 4 Institutes of Medical Biometry and Statistics, Faculty of Medicine and Medical Center, University of Freiburg, Freiburg, Germany; The University of Edinburgh, UNITED KINGDOM OF GREAT BRITAIN AND NORTHERN IRELAND

## Abstract

**Background:**

Colorectal cancer (CRC) remains a major global health challenge, with rapidly rising incidence in low- and middle-income countries, including Ethiopia. Survival outcomes are compromised by late-stage presentation, limited therapeutic capacity, and weak post-treatment follow-up. Although survival analysis is critical for characterizing CRC prognosis, insufficient attention has been given to the performance and validity of commonly applied survival models in Ethiopian populations, particularly when statistical assumptions are violated.

**Objective:**

To identify key clinical and treatment-related prognostic factors influencing overall survival among Ethiopian CRC patients and to assess the robustness and interpretability of Cox proportional hazard model (Cox PH model), stratified Cox proportional hazard model, frailty model, and Accelerated Failure Time (AFT) models, focusing on model adequacy under assumption violations.

**Methods:**

We conducted a retrospective cohort analysis of 161 colorectal cancer patients diagnosed between 2012 and 2016. Survival probabilities were estimated using Kaplan–Meier analysis, with group comparisons via log-rank tests. Multivariable modeling employed Cox proportional hazards, stratified Cox proportional hazard model, frailty, and accelerated failure time models. Model adequacy was evaluated through Akaike Information Criterion, concordance indices, and the Global Schoenfeld test for proportional hazards assessment.

**Results:**

The Cox proportional hazard model violated the proportional hazards assumption (global p = 0.043), primarily due to histological subtype effects, indicating that the standard Cox proportional hazard model was not appropriate for this dataset. Assumption violations were mitigated using the stratified Cox proportional hazard model (global p = 0.122). Among accelerated failure time models, the log-logistic distribution provided superior model fit (AIC = 649.88; concordance = 0.724). Time-ratio (TR) interpretation showed that surgery extended survival by approximately 2.4-fold, whereas elevated baseline carcinoembryonic antigen levels and metastatic disease reduced survival duration by nearly half. Elevated carcinoembryonic antigen, distant metastasis, vascular invasion, and squamous cell histology were consistently associated with poorer prognosis, while surgery and chemotherapy remained protective across models.

**Conclusion:**

Rigorous model checking indicates that the conventional Cox proportional hazards model is inappropriate for this cohort due to proportional hazard assumption violations. Accelerated failure time modeling, particularly the log-logistic form, offered improved fit and clearer time-based interpretation of covariate effects. These findings underscore the importance of model validation in CRC survival research and highlight the need for improved early detection, surgical access, and targeted management of aggressive tumor subtypes to enhance patient outcomes in Ethiopia.

## Introduction

Colorectal cancer (CRC) is a major contributor to global cancer-related morbidity and mortality, ranking as the third most commonly diagnosed malignancy and the second leading cause of cancer death worldwide [1]. While survival outcomes have gradually improved in high-income countries due to population-based screening, earlier detection, and expanded treatment options, low- and middle-income countries including Ethiopia continue to experience increasing incidence and persistently high mortality rates [2–9]. These trends are driven by late-stage diagnosis, restricted access to surgical and chemotherapeutic services, and fragmented follow-up systems [2–9].

In Ethiopia, hospital-based reports from tertiary oncology centers consistently show that most CRC patients present with advanced disease, often with regional or distant metastasis. Such clinical patterns result in poor survival outcomes and highlight the urgent need for stronger prognostic tools to support clinical decision-making and inform national cancer control strategies [2–9].

A rigorous evaluation of prognostic factors in colorectal cancer (CRC) requires analytical methods capable of capturing the substantial heterogeneity in disease progression and survival outcomes. Variations in tumour biology, stage at diagnosis, treatment response, and patient characteristics often result in complex and diverse survival trajectories that challenge conventional modelling approaches. The Cox proportional hazards (PH) model remains the most widely used framework for survival analysis because of its semi-parametric flexibility and straightforward interpretation of hazard ratios. However, its validity depends fundamentally on the proportional hazards assumption—that the effects of covariates remain constant over time [10,11]. In clinical practice, this assumption is frequently violated in heterogeneous patient populations, where the effects of prognostic factors may change during follow-up, potentially leading to biased estimates, reduced predictive performance, and misleading clinical inferences [12,13]. Alternative modelling approaches, including stratified Cox PH models, frailty models, and accelerated failure time (AFT) models, have been developed to accommodate non-proportional hazards, account for unobserved heterogeneity, and provide more robust estimates when the standard Cox PH assumptions are not satisfied [12–16]. Nevertheless, comparative evaluations of these approaches in CRC remain limited, and evidence on their relative performance for identifying prognostic factors and improving risk prediction is still scarce, highlighting the need for a comprehensive methodological assessment.

Despite the frequent application of the Cox model in Ethiopian CRC studies, previous research routinely assumes PHs but does not formally test this assumption [2]. If the PH assumption is violated as our preliminary diagnostic analysis demonstrates the Cox model yields biased estimates and misleading inference [12]. Therefore: The central research problem is that prior CRC survival studies in Ethiopia have used the Cox model without verifying its assumptions, leading to potentially invalid prognostic conclusions [2].

The aim of this study is to: identify key clinical and treatment-related prognostic factors affecting survival among Ethiopian CRC patients and to determine which survival modeling framework Cox PH model, stratified Cox PH model, frailty model, or AFT model provides the most reliable and interpretable results when statistical assumptions are violated.

By systematically comparing these models, this study evaluates how each method performs under real-world clinical heterogeneity, assesses robustness to assumption violations, and determines which approach provides the best prognostic accuracy and clinical interpretability. This research contributes essential methodological clarity to CRC survival analysis in Ethiopia and supports the development of more valid and context-appropriate prognostic tools in resource-limited settings.

## Data and methodology

### Study design and data source

A retrospective cohort study was conducted using anonymized, publicly accessible CRC patient data obtained from the Mendeley Data Repository [17]. The dataset consisted of 161 histologically confirmed colorectal cancer cases diagnosed between January 2012 and December 2016 in Ethiopia. The dataset collects from Addis Ababa population based cancer registry, from a tertiary level hospital Tikur Anbesa Specialized Hospital (TASH) which is equipped cancer diagnostic and treatment facilities and is one of the cancer treatment centers in Ethiopia [17]. Each patient was followed from the date of confirmed diagnosis until the date of death or end of observation. Patients who remained alive at the end of follow-up or were lost to follow-up were treated as censored observations [17]. Records were collected from TASH cancer registry using the medical record number. In case of absence, phone interview was done to all patients and/or their attendants on order to gather full information about the patient [17].

### Exclusion criteria

Patient records with neither histopathology report or cancer stage at diagnostic were excluded from the study. Out of 174 CRC patient charts retrieved, 13 (7.5%) were excluded due to no report of neither histopathology nor cancer stage [17].

### Study variables

#### Primary outcome.

The primary outcome was **overall survival (OS)**, defined as the time (in months) from the date of colorectal cancer diagnosis to the date of death from any cause or the end of follow-up, whichever occurred first.

- **Overall survival time (months):** Calculated as the interval between the date of diagnosis and the date of death or censoring.
- **Event indicator:** Coded as **1** for patients who died during the follow-up period and **0** for patients who were alive at the end of follow-up or lost to follow-up (censored).

#### Predictor variables.

The predictor variables included socio-demographic, clinical, and treatment-related characteristics.

#### Socio-demographic characteristics.

- Age at diagnosis (years)
- Sex (male/female)

#### Clinical characteristics.

- Tumour, Node, and Metastasis (TNM) stage
- Presence of distant metastasis (yes/no)
- Vascular invasion (yes/no)
- Histological subtype
- Baseline carcinoembryonic antigen (CEA) level

Baseline CEA levels were dichotomized as **normal (≤5 ng/mL)** and **elevated (>5 ng/mL)**, corresponding to CEA-negative and CEA-positive status, respectively, in accordance with recommendations from the National Comprehensive Cancer Network (NCCN) and the American Society of Clinical Oncology (ASCO) guidelines for colorectal cancer [18–20].

#### Treatment-related characteristics.

- Surgical resection (yes/no)
- Chemotherapy (yes/no)
- Radiotherapy (yes/no)

### Data management and quality control

Data were examined for completeness and consistency. Biologically implausible entries and duplicates were removed. Missing values were assessed; variables with <10% missingness were imputed using clinically relevant categorization or median replacement, while cases with incomplete survival time or unknown event status were excluded from analysis [3,4]. The percentage of missingness was 4.3% clinical trial stage, 2.5% smoking, 0.6% CEA, 5.6% family history, and 1.2% alcohol. Moreover, there was collinearity between clinical stage and TNM stage which leads to exclude clinical stage from the final model analysis. Missing values in CEA were replaced using the most frequent category because the proportion of missing data was minimal. Given the low level of missingness, the impact on model estimates was expected to be negligible [21,22].

### Variable selection and model building strategy

The full model included candidate variables. These variables were selected based on prior literature, clinical relevance, and availability in the dataset. Candidate variables with more missing values as well as indicating mullticollinearity excluded from the model. Then, with the remaining candidates a stepwise variable selection was conducted. All survival models were used the selected predictor variables.

### Statistical analysis

Survival distributions were estimated using the Kaplan–Meier method, and differences between groups were evaluated with log-rank tests. Median survival times with 95% confidence intervals were reported where applicable [4].

### Multivariable survival models

Four modeling frameworks were applied: cox PH model [10,23], cox PH assumption and stratified model [24], Frailty model [25], and AFT model [14].

**Cox PH model**: assuming constant hazard ratios over time.

$h\left(t|X\right)=h_{\text{0}}(ttextrmexp(\beta^{T}X)$, Where $h_{\text{0}}(t)$ is hazard baseline function, X observed value, t time. Hazard is proportional over time; β interpreted using hazard ratios [10,11].

**Stratified Cox PH model**: used when categorical covariates violated the PH assumption, allowing baseline hazards to vary by stratum while estimating common covariate effects.

$h_{k}\left(t|X\right)=h_{\text{0}k}(ttextrmexp(\beta^{T}X)$, Allows distinct baseline hazards $h_{\text{0}k}$ across strata k [24].

**Frailty model**: incorporating a random effect term to account for unobserved heterogeneity (e.g., clustering by hospital/region when applicable)- we apply region cluster in our study.

$h\left(t|X,\;u\right)=h_{\text{0}}(ttextrmexp(\beta^{T}X+u)$, Frailty term u captures unobserved heterogeneity or clustering [25].

**AFT models**: including log-normal, Weibull, and log-logistic distributions. The AFT model expresses covariate effects as time ratios, facilitating time-based interpretation of survival outcomes [12–14,26].

$Log(T)=\;\beta^{T}X\;+\;\sigma \varepsilon$, Models survival time directly; effects interpreted using time ratios [12–14].

### Model assumption testing and evaluation

- PH assumption was assessed using Schoenfeld residuals and the Global Schoenfeld test [10].
- Model fit was compared using Akaike Information Criterion (AIC) and concordance index (C-index) – to compare two different models stratified model and AFT model.
- Model significance was evaluated using likelihood ratio, Wald, and score tests.

Predictor selection followed backward stepwise elimination guided by statistical significance and clinical relevance. A p-value < 0.05 was considered statistically significant.

### Software

All statistical analyses were conducted using R (version 4.5.1) with relevant packages.

### Ethical considerations

The study utilized publicly available, secondary data with no identifiable patient information. All analyses adhered to principles of confidentiality and responsible data use.

Data source: https://data.mendeley.com/datasets/vvzw3wkx93/3?utm_source=chatgpt.com

## Results and discussions

### Descriptive survival patterns and Kaplan–Meier estimates

Table 1 shows the baseline of the socio-demographic characteristics of patients with colorectal cancer. A total of 161 patients with colorectal cancer were included in the study. As Table 1 shows, the mean age at diagnosis was 45.86 years, indicating that colorectal cancer primarily affected middle-aged and older adults. Males accounted for 63.35% of the study population, while females represented 36.65%. Most patients were single (78.9%) and had secondary school (35.4%). A family history of colorectal cancer was reported in 2.48% of patients. Alcohol consumption and tobacco use were reported by 8.07% and 6.83% of patients, respectively.

**Table 1:** Baseline socio-demographic characteristics of patients with colorectal Cancer Patients.

Characteristic	0 N = 86^1^	1 N = 75^1^	p-value^2^
Age	45 (35, 58)	45 (32, 58)	0.7
Sex			0.6
Female	33 (38%)	26 (35%)	
Male	53 (62%)	49 (65%)	
Region			0.031
Addis Ababa	52 (60%)	30 (40%)	
Amhara	8 (9.3%)	16 (21%)	
Diredawa	1 (1.2%)	1 (1.3%)	
Hareri	1 (1.2%)	1 (1.3%)	
Oromia	11 (13%)	20 (27%)	
SNNPR	8 (9.3%)	3 (4.0%)	
Somali	1 (1.2%)	1 (1.3%)	
Tigray	4 (4.7%)	3 (4.0%)	
Marital Status			0.094
Divorced	0 (0%)	1 (1.3%)	
Married	12 (14%)	19 (25%)	
Single	73 (85%)	54 (72%)	
Widowed	1 (1.2%)	1 (1.3%)	
Educational status			0.4
Higher Education	12 (14%)	8 (11%)	
No formal education	22 (26%)	22 (29%)	
Primary level	25 (29%)	15 (20%)	
Secondary level	27 (31%)	30 (40%)	
Family history (Yes)	2 (2.3%)	2 (2.7%)	>0.9
Alcohol (Yes)	6 (7.0%)	7 (9.3%)	0.6
Tobacco (Yes)	4 (4.7%)	5 (6.7%)	0.7
Location			0.2
Anorectal	2 (2.3%)	1 (1.3%)	
Colon	63 (73%)	46 (61%)	
Recto Sigmoid Junction	9 (10%)	8 (11%)	
Rectum	12 (14%)	20 (27%)	

^1^ Median (Q1, Q3); n (%)

^2^ Wilcoxon rank sum test; Pearson’s Chi-squared test; Fisher’s exact test

Table 2 Presents the baseline clinical and treatment characteristics of patients with colorectal cancer. The majority of tumors were located in the colon (67%). Adenocarcinoma was the predominant histological type (96.89%). Most tumors were well differentiated (55.9%), followed by moderately differentiated and poorly differentiated tumors. At diagnosis, a substantial proportion of patients presented with advanced disease. Stage IV disease accounted for 47.82% of cases. With respect to treatment, chemotherapy was administered to 83.85% of patients, surgery was performed in 15.15%, and radiotherapy was received by 6.2%. The median follow-up time was 14.00 months. During follow-up, 75 patients (46.58%) died, while 86 patients (53.42%) were censored.

**Table 2:** Baseline clinical and treatment characteristics of patients with colorectal cancer.

Characteristic	0 N = 86^1^	1 N = 75^1^	p-value^2^
Location			0.2
Anorectal	2 (2.3%)	1 (1.3%)	
Colon	63 (73%)	46 (61%)	
Recto Sigmoid Junction	9 (10%)	8 (11%)	
Rectum	12 (14%)	20 (27%)	
Histological type			0.020
Adenocarcinoma	86 (100%)	70 (93%)	
Squamous cell carcinoma	0 (0%)	5 (6.7%)	
Histological grade			0.021
Anaplastic	1 (1.2%)	0 (0%)	
Moderately differentiated	17 (20%)	19 (25%)	
Poorly differentiated	12 (14%)	22 (29%)	
Well differentiated	56 (65%)	34 (45%)	
TNM_stage			<0.001
Stage I	4 (4.7%)	1 (1.3%)	
Stage II	41 (48%)	16 (21%)	
Stage III	13 (15%)	9 (12%)	
Stage IV	28 (33%)	49 (65%)	
Clinical_stage			<0.001
Localized	25 (29%)	9 (12%)	
Locally advanced	31 (36%)	18 (24%)	
Metastasis	30 (35%)	48 (64%)	
CEA_baseline (Elevated)	30 (35%)	52 (69%)	<0.001
Dist_metastasis (Yes)	31 (36%)	50 (67%)	<0.001
Vascular Invasion (Yes)	1(1.2%)	4(5.3%)	0.2
Chemotherapy (Yes)	73 (85%)	62 (83%)	0.7
Surgery (Yes)	21 (24%)	4 (5.3%)	<0.001
Radiotherapy (Yes)	3 (3.5%)	7 (9.3%)	0.2

^1^ Median (Q1, Q3); n (%)

^2^ Wilcoxon rank sum test; Pearson’s Chi-squared test; Fisher’s exact test

Fig 1 survival curve compares overall survival among patients categorized by TNM Stage (I–IV) over approximately 60 months (5 years) of follow-up. Patients with Stage I show the best prognosis, maintaining the highest survival probabilities throughout the follow-up period. Stage II survival is lower than Stage I but still relatively favorable. Stage III shows a noticeable drop, especially early in follow-up. Stage IV patients have the poorest survival, with rapid decline in the first 20–30 months. This means the variation in survival across TNM stages is highly significant, not due to chance. (p-value = 1e-04 (< 0.001). Event patterns: the steeper the slope, the higher the event (death) rate. Stage IV curve drops sharply early on suggests advanced disease stage strongly predicts early mortality. Censoring occurs across stages but more at later time points, showing ongoing follow-up for some patients.

**Fig 1:**
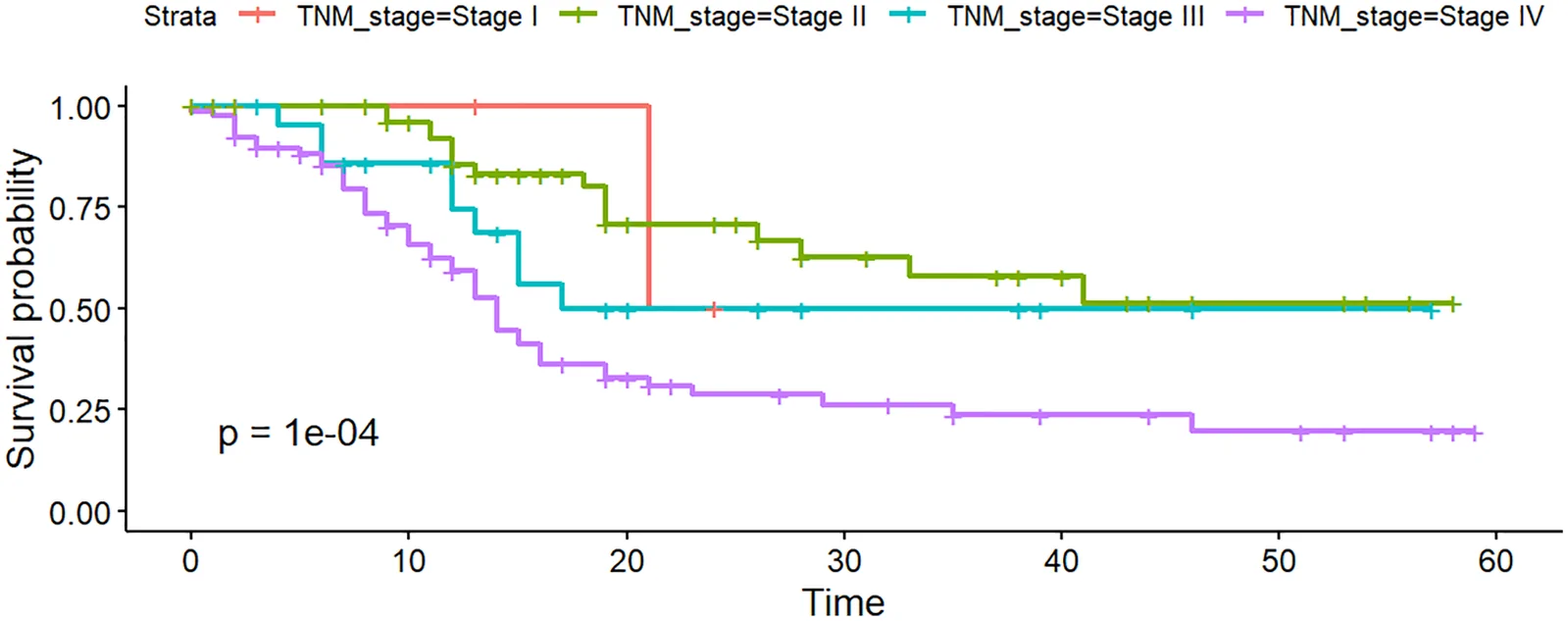
Kaplan–Meier overall survival curves stratified by TNM stage among patients with colorectal cancer.

Table 3 displays log-rank test of survival differences of CRC by TNM-stages. A chi-square test demonstrated a statistically significant difference in death among the four TNM-stages (Chi-square = 21.1, df = 3, p = 0.0001). TNM-stage II had fewer observed deaths than expected, whereas TNM-stage IV shows higher than expected death which is worse survival stage. TNM-stage I and III showed minor deviations from expected values. These findings indicate that CRC death is significantly associated with TNM-stage membership, with the greatest differences observed in TNM-stages II and IV.

**Table 3:** Log-rank test comparing overall survival across TNM stages among patients with colorectal cancer.

TNM-Stage	N	Observed (O)	Expected (E)	(OE)^2^/E	(OE)^2^/V
Stage I	5	1	2.27	0.713	0.765
Stage II	57	16	31.73	7.800	14.027
Stage III	22	9	10.69	0.266	0.320
Stage IV	77	49	30.31	11.528	20.056

O = Observed value, E = Expected value, Chi-square = 21.1 df = 3 p = 0.0001 Significant survival differences exist across TNM stages (p = 0.0001).

### Cox proportional hazards model

Table 4 the conventional Cox PH model, several predictors were associated with a statistically significant increase in the hazard of death. Elevated baseline carcinoembryonic antigen (CEA) levels (HR = 2.07) and the presence of distant metastasis at diagnosis (HR = 2.29) both nearly doubled the hazard of mortality. Vascular invasion was associated with a tripling of death risk (HR = 3.01), while squamous cell histology exhibited the most aggressive behavior (HR = 6.01), indicating substantially poorer prognosis relative to adenocarcinoma.

**Table 4:** Multivariable Cox proportional hazards (PH) regression analysis of prognostic factors associated with overall survival among patients with colorectal cancer.

Characteristic	Coef	HR (exp(Coef))	se(Coef)	95% CI	p-value
CEA baseline (elevated)	0.73	2.07	0.29	1.18, 3.64	0.011
Surgery (Yes)	−1.39	0.25	0.53	0.09, 0.70	0.008
Distance metastasis (Yes)	0.83	2.29	0.27	1.34, 3.93	0.003
Histology type (Squamous cell carcinoma)	1.79	6.01	0.50	2.26, 16.0	<0.001
Chemotherapy (Yes)	−0.72	0.49	0.32	0.26, 0.91	0.025
Vascular invasion (Yes)	1.10	3.01	0.54	1.05, 8.62	0.041

Abbreviations: Coef= Coefficient, CI = Confidence Interval, HR = Hazard Ratio.

Conversely, treatment effects were protective: surgical resection was associated with a 75% reduction in the hazard of death (HR = 0.25), while chemotherapy was associated with a 51% reduction (HR = 0.49).

However, Table 5 and Fig 2, the global Schoenfeld residual test indicated violation of the PH assumption (chisq-X^2^ = 13.002, p = 0.043), driven primarily by the histological subtype variable (P-value = 0.043*). This suggests that hazard ratios were not constant over time, thereby undermining the validity of the standard Cox model for this dataset.

**Table 5:** Results of the Schoenfeld residual tests for assessing the proportional hazards assumption of the Cox proportional hazards model.

Characteristic	Chisq	df	p-value
CEA baseline	2.493	1	0.114
Surgery	0.763	1	0.382
Distance metastasis	1.232	1	0.267
Histology type	5.565	1	0.018
Chemotherapy	0.925	1	0.336
Vascular invasion	1.613	1	0,204
GLOBAL	13.002	6	0.043

Chisq= chi-square, df= degree of freedom.

**Fig 2:**
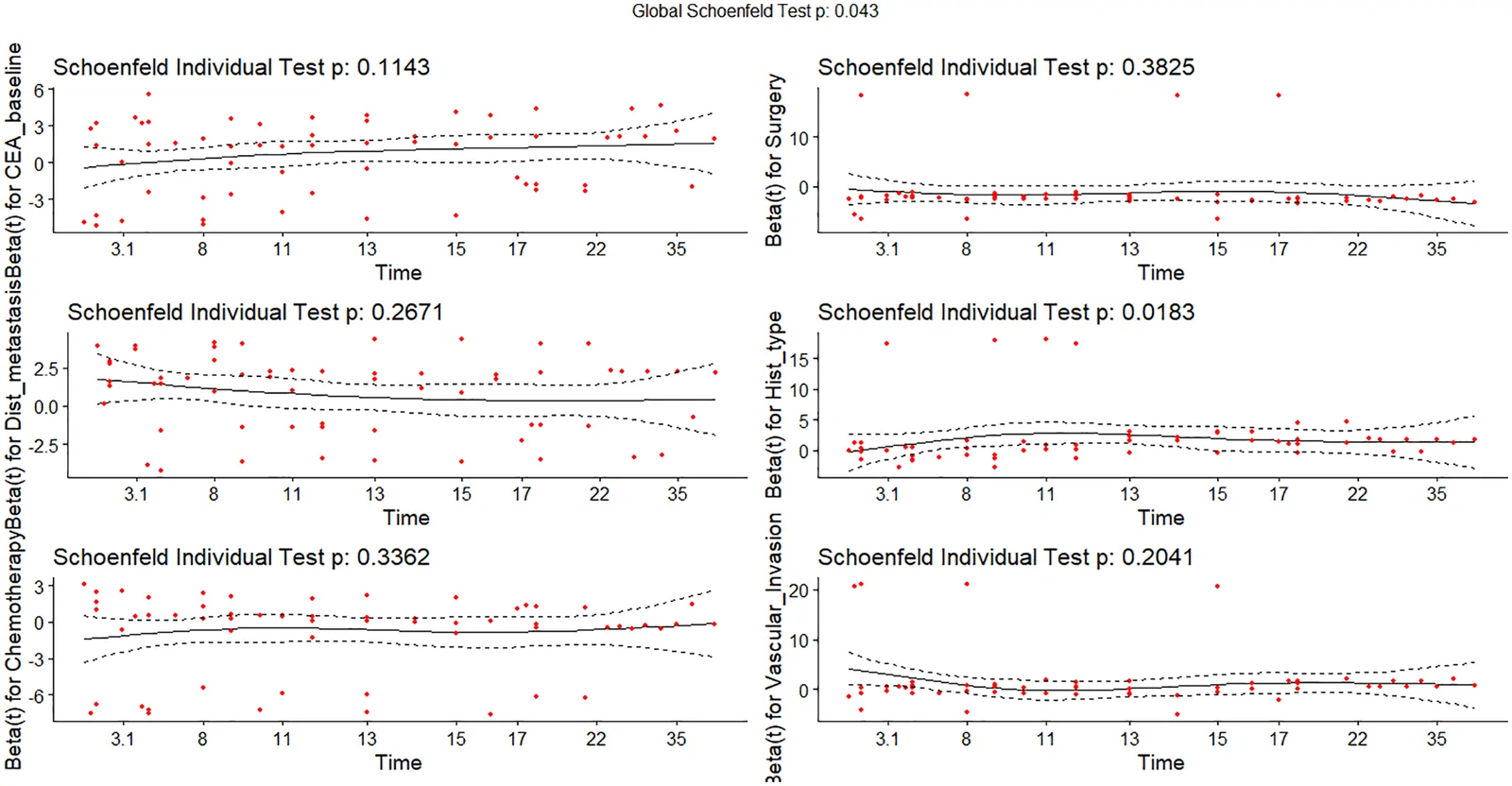
Schoenfeld residual plots for assessing the proportional hazards assumption of the Cox proportional hazards model.

Fig 2 Schoenfeld residual plots for histological subtype indicated deviation from the proportional hazards assumption, with evidence of time-varying effects. Individual tests are presented for each covariate, together with the global Schoenfeld test. The global test indicated evidence of a violation of the proportional hazard’s assumption (P  =  0.043), primarily attributable to the histological subtype variable (P  =  0.018).

### Stratified Cox PH model

To account for the violation of the proportional hazards (PH) assumption observed in the standard Cox model, a stratified Cox proportional hazards model was fitted using histological subtype as the stratification variable. The model identified several clinic-pathological factors that were independently associated with overall survival. Elevated baseline CEA levels were associated with a two-fold increased hazard of death (HR = 2.01, 95% CI: 1.13–3.56, *P* = 0.017), while the presence of distant metastasis significantly increased the hazard of death (HR = 2.31, *P* = 0.003). Conversely, surgical resection (HR = 0.26, 95% CI: 0.09–0.75, *P* = 0.012) and chemotherapy (HR = 0.51, 95% CI: 0.27–0.96, *P* = 0.040) were associated with significantly improved survival. Although vascular invasion was associated with an increased hazard of death (HR = 2.76), the association did not reach statistical significance (*P* = 0.060).

The stratified Cox model demonstrated moderate predictive discrimination with a concordance index of **0.633** (SE = 0.041) Table 6, 7. The overall model was statistically significant based on the likelihood ratio test (χ^2^ = 21.4, df = 5, *P* < 0.001), indicating that the included covariates collectively explained a significant proportion of the variation in survival.

**Table 6:** Results of the multivariable stratified Cox proportional hazards regression model for prognostic factors associated with overall survival among patients with colorectal cancer.

Characteristic	Coef	HR (exp(Coef))	se(Coef)	95% CI	p-value
CEA baseline (elevated)	0.69	2.01	0.29	1.13, 3.56	0.017
Surgery (Yes)	−1.33	0.26	0.53	0.09, 0.75	0.012
Distance metastasis (Yes)	0.84	2.31	0.59	0.72, 7.39	0.003
Chemotherapy (Yes)	−0.67	0.51	0.32	0.27, 0.96	0.04
Vascular Invasion (Yes)	1.02	2.76	0.54	0.96, 7.96	0.06

Abbreviations: Coef=Coefficient, CI = Confidence Interval, HR = Hazard Ratio

Concordance= 0.633 (se = 0.041), Likelihood ratio test= 21.4 on 5 df, p=7e-04

**Table 7:** Schoenfeld residual tests for assessing the proportional hazards assumption of the stratified Cox proportional hazards model.

Characteristic	chisq	df	p
Surgery	0.5738	1	0.449
CEA baseline	4.8690	1	0.027
Chemotherapy	0.8060	1	0.369
Vascular Invasion	1.0681	1	0.301
Distance metastasis	0.0498	1	0.823
GLOBAL	6.4325	5	0.266

#### Assessment of the proportional hazards assumption.

The proportional hazards assumption for the stratified Cox model was evaluated using Schoenfeld residuals. The **global Schoenfeld test was not statistically significant** (χ^2^ = 6.43, df = 5, *P* = 0.266), indicating no evidence of an overall violation of the proportional hazards assumption after stratification by histological subtype. Most covariates satisfied the PH assumption (*P* > 0.05). However, baseline CEA remained statistically significant (*P* = 0.027), suggesting residual evidence of non-proportional hazards for this variable. Nevertheless, the absence of a significant global test indicates that the stratified Cox model adequately addressed the overall violation observed in the standard Cox model and provided an acceptable framework for estimating covariate effects.

### Shared frailty model

To account for potential unobserved heterogeneity among patients from different regions, a shared frailty Cox proportional hazards model with a Gaussian random effect for region was fitted. The estimated frailty variance was extremely small (variance = 5 × 10^−7^) and was not statistically significant (*P* > 0.90), indicating negligible between-region variability in survival after adjustment for the measured covariates. This finding suggests that regional clustering did not contribute meaningfully to unexplained differences in mortality risk.

The estimated hazard ratios for the fixed effects remained virtually unchanged compared with those obtained from the standard Cox proportional hazards model. Elevated baseline CEA level (HR = 2.07, 95% CI: 1.18–3.64, *P* = 0.011), distant metastasis (HR = 2.29, 95% CI: 1.34–3.93, *P* = 0.003), vascular invasion (HR = 3.01, 95% CI: 1.05–8.62, *P* = 0.041), and squamous cell carcinoma histology (HR = 6.01, 95% CI: 2.26–16.0, *P* < 0.001) were associated with an increased hazard of death, whereas surgical resection (HR = 0.25, 95% CI: 0.09–0.70, *P* = 0.008) and chemotherapy (HR = 0.49, 95% CI: 0.26–0.91, *P* = 0.025) were associated with improved survival.

Overall, the shared frailty model did not provide evidence that incorporating regional random effects improved model fit or altered the estimated effects of the prognostic factors. These findings indicate that unmeasured regional-level frailty had little influence on overall survival, and that the standard Cox model adequately captured the observed variation in survival, Table 8.

**Table 8:** Results of the shared frailty Cox proportional hazards model with Gaussian random effects for region, evaluating prognostic factors associated with overall survival among patients with colorectal cancer.

Characteristic	HR	95% CI	p-value
CEA baseline (elevated)	2.07	1.18, 3.64	0.011
Surgery (Yes)	0.25	0.09, 0.70	0.008
Distance metastasis (Yes)	2.29	1.34, 3.93	0.003
Chemotherapy (Yes)	0.49	0.26, 0.91	0.025
Vascular invasion (Yes)	3.01	1.05, 8.62	0.041
Histology type (Squamous cell carcinoma)	6.01	2.26, 16.0	<0.001
frailty(Region)			>0.9

Abbreviations: CI = Confidence Interval, HR = Hazard Ratio

Variance of random effect= 5e-07 I-likelihood = -308.7

### Accelerated Failure Time (AFT) modeling

#### Model selection and goodness-of-fit.

To identify the most appropriate parametric survival model for colorectal cancer (CRC), three accelerated failure time (AFT) distributions including Weibull, log-normal, and log-logistic were evaluated using the Akaike Information Criterion (AIC). Among the candidate models, the log-logistic AFT model demonstrated the best overall fit, yielding the lowest AIC (649.88), followed by the log-normal (AIC = 650.73) and Weibull (AIC = 655.37) models (Table 9). The lower AIC indicates that the log-logistic model achieved the optimal balance between model fit and complexity.

**Table 9:** Comparison of accelerated failure time (AFT) models using the Akaike Information Criterion (AIC) for model selection among patients with colorectal cancer.

Model	Degrees of Freedom	AIC
AFT Weibull	8	655.37
AFT Log-Normal	8	650.73
AFT Log-Logistic	8	649.88

AIC = Akaike Information Criterion.

In addition to its superior goodness-of-fit, the log-logistic AFT model exhibited the highest predictive discrimination, with a concordance index of 0.724, exceeding that of the stratified Cox proportional hazards model (C = 0.633). These findings suggest that the log-logistic AFT model provided a more accurate representation of the survival experience of patients with colorectal cancer and offered improved prognostic performance compared with the Cox-based modelling approaches evaluated in this study.

Table 10 Log-logistic AFT model demonstrated good overall performance and was statistically significant (Likelihood Ratio χ^2^ = 45.88, df = 6, p < 0.001), with a concordance statistic of 0.724, indicating acceptable discriminative ability. The model identified both protective and harmful prognostic factors that significantly influenced survival time among colorectal cancer patients.

**Table 10:** Multivariable log-logistic accelerated failure time (AFT) model evaluating prognostic factors associated with overall survival among patients with colorectal cancer.

Variable	Coef	TR	Se (Coef)	Z	95% CI (lower, upper)	P-value
(Intercept)	3.4038		0.2662	12.79	(17.85, 50.68)	<2x10^-16^
CEA baseline (elevated)	−0.5990	0.55	0.2131	−2.81	(0.36, 0.83)	0.0049
Surgery (yes)	0.8674	2.38	0.3917	2.21	(1.1, 5.13)	0.0268
Distance metastasis (yes)	−0.5819	0.56	0.2107	−2.76	(0.36, 0.84)	0.0057
Chemotherapy (yes)	0.5106	1.67	0.2506	2.04	(1.02, 2.72)	0.0416
Vascular invasion (yes)	−1.2543	0.29	0.5145	−2.44	(0.1, 0.78)	0.0148
Histologic type (Squamous) (yes)	−1.0647	0.34	0.3984	−2.67	(0.16, 0.75)	0.0075
Log scale	−0.5871		0.0948	−6.19	(0.46, 0.67)	5.9x10^-10^

TR = Estimated Time Ratio, CI = Confidence interval, Scale = 0.556 Loglik(model)=−316.9Loglik (intercept only) =−339.9 Chisq = 45.88 on 6 degrees of freedom, p = 3.1e-08 Concordance = 0.724.

#### Protective factors associated with prolonged survival.

Receipt of surgery and chemotherapy emerged as significant protective factors. Patients who underwent surgical treatment experienced substantially longer survival times compared with those who did not receive surgery. The estimated time ratio (TR) for surgery was 2.38, indicating that survival time was approximately 2.4 times longer among surgically treated patients. Similarly, chemotherapy was associated with a significant survival benefit, increasing survival time by 67% (TR = 1.67). These findings highlight the critical role of timely and appropriate therapeutic interventions in improving colorectal cancer outcomes.

#### Harmful factors associated with reduced survival.

Several disease-related characteristics were associated with significantly shorter survival times. Elevated baseline carcinoembryonic antigen (CEA) levels reduced survival time by approximately 45% (TR = 0.55), suggesting that increased tumor burden or biological aggressiveness adversely affects prognosis. Patients presenting with distant metastasis experienced a 44% reduction in survival time (TR = 0.56), reflecting the unfavorable prognosis associated with advanced-stage disease.

Pathological indicators of aggressive tumor behavior were also important predictors of poor survival. Vascular invasion showed the strongest negative effect, reducing survival time by approximately 71% (TR = 0.29). Likewise, patients with squamous cell histology had a 66% shorter survival time than those with the reference histological subtype (TR = 0.34), indicating a markedly more aggressive disease course.

The findings suggest that survival among colorectal cancer patients is strongly influenced by a balance between treatment-related and disease-related factors. While surgery and chemotherapy substantially prolonged survival, elevated CEA levels, metastatic disease, vascular invasion, and squamous histology were associated with markedly shortened survival times. These results emphasize the importance of early diagnosis before metastatic spread, careful assessment of pathological risk factors, and ensuring access to effective treatment modalities. Furthermore, the AFT framework provides clinically meaningful estimates in terms of survival time ratios, allowing direct quantification of how specific factors accelerate or decelerate patient survival.

## Discussion

Colorectal cancer (CRC) remains one of the leading causes of cancer-related morbidity and mortality worldwide, underscoring the need for robust prognostic models to guide clinical decision-making. Although the Cox proportional hazards (PH) model is the most widely used approach for analyzing survival outcomes, its validity depends on key assumptions, particularly the proportional hazards assumption, independence of survival times, and appropriate handling of censoring. Failure to evaluate these assumptions may result in biased effect estimates, reduced predictive accuracy, and misleading clinical inferences. Despite the widespread application of survival models in CRC research, assessment and reporting of model assumptions remain inadequate in many studies. Therefore, this study systematically evaluated the assumptions underlying several commonly used survival models and compared their performance in identifying prognostic factors for overall survival among patients with CRC. Our findings demonstrated that the standard Cox PH model violated the proportional hazards assumption, whereas the stratified Cox model largely resolved this violation. However, among all competing approaches, the log-logistic accelerated failure time (AFT) model provided the best overall performance, achieving the lowest Akaike Information Criterion (AIC) and the highest predictive discrimination, indicating that it was the most appropriate model for the present data.

The final models included six estimated parameters for the Cox PH model, five for the stratified Cox model, seven for the frailty model, and seven for the log-logistic AFT model. Based on the observed number of events, the corresponding events-per-variable (EPV) ratios were approximately 12.5, 15.0, 10.7, and 10.7, respectively. The selected AFT model therefore satisfied the widely accepted recommendation of at least 10 events per parameter, suggesting a low risk of substantial overfitting [23]. Furthermore, sensitivity analyses using simplified models yielded similar effect estimates, with no meaningful changes in the direction or magnitude of the principal associations, providing additional evidence for the robustness and stability of the study findings [23].

An important finding of this study was that the proportional hazards assumption was violated primarily for histological subtype, indicating that the effect of tumor histology on mortality was not constant over time [2]. This finding suggests that certain histological subtypes may exert a stronger influence on survival during the early stages following diagnosis, with their impact diminishing as follow-up progresses. One possible explanation is that more aggressive histological variants, such as squamous cell carcinoma, are characterized by rapid tumor growth, greater invasive potential, earlier metastatic spread, and reduced responsiveness to standard treatment modalities, resulting in a substantially elevated risk of death shortly after diagnosis. However, among patients who survive the initial high-risk period, the relative effect of histological subtype may decrease because long-term survivors tend to represent a selected subgroup with more favorable biological characteristics or better treatment response. In addition, as follow-up lengthens, other factors such as treatment effects, disease recurrence, comorbid conditions, and survivorship dynamics may play a greater role in determining outcomes, thereby attenuating the influence of histology on mortality risk. These findings highlight the complex and time-varying nature of the relationship between histological subtype and survival in colorectal cancer and leads for the use of the AFT model, which accommodates such non-proportional effects and offers clinically interpretable estimates in terms of changes in survival time [24,27,28].

The time-varying effect of histology suggests that the relative risk between histological subtypes is stronger in early follow-up and diminishes over time, which may reflect differences in early disease aggressiveness or treatment response. In the stratified Cox model, histology was used as a stratification variable, allowing for separate baseline hazard functions. While this approach resolves the proportional hazards violation, it precludes direct estimation of a hazard ratio for histology [24,27,28].

Based on the selected AFT model, the present study identified several clinical and treatment-related variables that significantly influenced survival outcomes among Ethiopian colorectal cancer patients. Elevated baseline carcinoembryonic antigen (CEA) and distant metastasis emerged as strong predictors of mortality reinforcing their role as indicators of advanced disease severity. These findings are consistent with prior Ethiopian analyses, including [2–6]. Carcinoembryonic antigen level ≥5ng/ml [AHR = 1.84; 95% CI: (1.02, 3.30) which is consistent with [3]. Metastatic cancer were 4.221 times likely to die compared to non-metastatic patients [6] which similarly demonstrated that tumor burden and biomarker elevation correlate with decreased survival. Internationally, analogous relationships between metastatic spread, biomarker elevation, and poor outcomes have been widely documented in foundational and contemporary studies, such as [15], confirming the external validity of our results.

Surgical resection and chemotherapy demonstrated substantial survival benefits in this cohort, with patients who underwent surgery experiencing a more than two-fold extension of survival relative to those who did not. This aligns with evidence reported by [5,7], underscoring the critical role of surgical intervention and systemic therapy in improving post-diagnosis longevity. These results emphasize the urgency of early surgical eligibility through timely detection and referral, particularly in a healthcare system where delayed presentation is common.

A central methodological contribution of this study lies in its assessment of model-specific assumptions. The violation of the proportional hazard assumption in the Cox PH model, particularly regarding histological subtype, indicates that the effect of tumor histology on risk is not constant over time. Such temporal variability has been emphasized in theoretical discussions of survival modeling by Cox (1972) [10] and by diagnostic analyses from [11]. By implementing a stratified Cox PH model, this issue was effectively mitigated, confirming that stratification by histology yields valid parameter estimates while retaining interpretive value.

Importantly, the use of AFT modeling particularly the log-logistic form provided superior model performance and enabled clinically intuitive time-based interpretation. Similar methodological advocates of AFT approaches in cancer survival include [12,13], who reported improved interpretability and robustness when PH assumptions are not met. The current findings substantiate these insights, demonstrating that AFT outputs such as time ratios convey more tangible prognostic meaning for clinicians and policymakers.

The frailty model indicated no significant unobserved heterogeneity across regions, suggesting relatively homogeneous clinical management or disease progression across centers. This finding is congruent with the conclusions of [6], who likewise observed minimal regional variability in colorectal cancer (CRC) survival outcomes in Ethiopia.

Taken together, our findings demonstrate that clinical and pathological prognostic indicators remain dominant predictors of survival in CRC, while also establishing those analytic conclusions are sensitive to model specification. The divergence in model adequacy specifically the inadequacy of Cox PH without assumption verification highlights a critical methodological oversight in prior Ethiopian colorectal cancer (CRC) research [2]. By rigorously testing assumptions and implementing alternative modeling frameworks, this study provides evidence that AFT modeling offers superior fit and interpretability for this population [24,27].

**Strengthen of the Study:** This study assessed and evaluated assumptions of survival models in CRC patient dataset. Moreover, model comparison was conducted on those models fully satisfied methodological and statistical assumptions using goodness of fit test metrics. The finding provides evidence-based result which integrated statistical and oncological methods.

**Study limitations:** include the modest sample size and reliance on secondary retrospective data, which may limit external generalizability and preclude assessment of certain behavioral or genetic risk factors not captured in the dataset. However, the multicenter composition of the sample and methodological rigor in model comparison strengthens internal validity.

## Conclusion

This study demonstrates that evaluating multiple survival modeling approaches improves the robustness of prognostic assessment in patients with colorectal cancer. Among the models examined, the log-logistic accelerated failure time (AFT) model provided the best overall fit and predictive performance, while the stratified Cox proportional hazards model offered a valid alternative when the proportional hazards assumption was violated. Elevated baseline carcinoembryonic antigen (CEA), distant metastasis, squamous cell histology, and vascular invasion were associated with poorer survival, whereas surgery and chemotherapy significantly improved survival outcomes. These findings highlight the importance of selecting appropriate survival models based on their underlying assumptions and reinforce the need for early diagnosis and timely access to effective treatment to improve colorectal cancer outcomes in Ethiopia. Future multicentre studies with larger cohorts are warranted to validate these findings and enhance prognostic modelling across diverse populations.

### Recommendations

Based on these findings, several targeted recommendations emerge for clinical practice and future research:

- Strengthen CRC awareness and education programs to improve recognition of early symptoms and encourage timely healthcare-seeking behavior among high-risk populations.
- Improve access to early diagnosis by expanding screening services, enhancing diagnostic capacity, and streamlining referral pathways to minimize delays in diagnosis.
- Increase timely access to surgical treatment and adjuvant chemotherapy by strengthening multidisciplinary cancer care, reducing waiting times, and improving the availability of specialized oncology services.
- Address health system and socioeconomic barriers, including financial constraints, transportation challenges, and inequitable access to specialized care, through targeted health polices and patient support programs.
- Implement regular carcinoembryonic antigen surveillance as part of standardized follow-up protocols, particularly for patients at elevated risk of recurrence.
- Employ AFT based analytical methods in future studies where proportional hazard assumptions are suspect or demonstrably violated.
- Expand research toward multicenter and longitudinal cohorts to enhance external validity and capture regional heterogeneity in colorectal cancer (CRC) presentation and treatment outcomes.

## Abbreviations

CRC: colorectal cancer
AFT: Accelerated Failure Time
TR: time ratio
CEA: carcinoembryonic antigen
PH: proportional hazard
C-index: concordance index
AIC: Akakie Information Criteria
