# Supplementary material for: Survival modeling and assessing prognostic factors of colorectal cancer in Ethiopia: Addressing violation of statistical assumptions
Source: PLoS One. 2026 Oct 26;21(10):e0361233. doi: 10.1371/journal.pone.0361233 (PMC13705600; doi:10.1371/journal.pone.0361233)
Supplement: Supplementary file 1 [file pone.0361233.s001.docx]

Comments on manuscript titled: Survival Modeling and Assessing Prognostic Factors of Colorectal Cancer in Ethiopia: Addressing Violation of Statistical Assumptions

Comments

Strength

The manuscript is well organized and written in a Standard English language. It is also easily understandable.

Issues observed that need keen attention of the author

1. The term region and hospital cannot go together. It is better to use the term hospital. Most importantly since the author said he applied region cluster, it would be better if it could be hospital, he should show the patients by region to see how many patients were treated in which region. Because one of the issue raised by the author is absence early detection and urgency of early surgical. These issues are related to the region and the hospital level due to far away from the capital city of the country.
2. Since the number of patients included in the study is too small, it is difficult to generalize at national level.
3. At the head of the manuscript, the name of one author only is mentioned but in the body of the manuscript, he said ‘we’. If there are other authors, their names should be mentioned otherwise the word ‘we’ should be replaced by ‘I’.
4. The author said in the manuscript that the patients were followed up until death. But the author said nothing how he knew these patients were followed up until their death and he relayed on the processed retrospective data only and he did not refer the patients’ charts and interviewed the health professionals where the patients were treated. Thus, it is difficult to say that the patients were followed up until death as there might be patients who were not followed up for various reasons.
5. The results are too general and generic. The discussion part is too shallow and was not linked with other studies as well as the results. It also lack interpretation.
6. The recommendations are too generic and does not consider other dimensions that prohibit the early detection and early surgery.
7. One result that impressed me was “the standard Cox model was not appropriate for this dataset” which was mentioned in the abstract but not indicated in the result section. This result was interesting and I was expecting the author to say something how such issues can be handled. However, the author did not say anything. I encourage the author to see this result and come up with new idea as this will be the value add of this manuscript.
8. References are too few. I kindly encourage the author to read more references that will enable him to analyze and interpret his data.
