# Supplementary material for: Survival modeling and assessing prognostic factors of colorectal cancer in Ethiopia: Addressing violation of statistical assumptions
Source: PLoS One. 2026 Oct 26;21(10):e0361233. doi: 10.1371/journal.pone.0361233 (PMC13705600; doi:10.1371/journal.pone.0361233)
Supplement: Supplementary file 2 [file pone.0361233.s002.docx]

Response to Reviewer comments

**Journal:** PLOS One

**Submission ID:** PONE-D-26-16119

**Topic:** Survival Modeling and Assessing Prognostic Factors of Colorectal Cancer in Ethiopia: Addressing Violation of Statistical Assumptions

**Authors:** Tuem Gebre Abraha (TGA), Mulu Reda Terefe (MRT), Zeytu Gashaw Asfaw (ZGA)

**Dear Editor-in-chief,**

We sincerely appreciate you and the reviewer for taking the time to read our article and provide thoughtful feedback. Your comments have been invaluable in improving our manuscript. We have carefully considered each point raised and made every effort to address them thoroughly. We hope that we have addressed all the reviewers’ concerns and that the manuscript is now ready for publication.

**Dear Respected Reviewer**

We sincerely thank you for your thorough and constructive feedback. We have carefully addressed all the comments and concerns raised, ensuring a more cautious interpretation of the findings and improving the overall clarity of the manuscript. In addition, we have refined the language, corrected grammatical and spelling errors, reference formatting and citation consistency, and strengthened the presentation of the content.

Our point-by-point responses are provided below, and most of the revisions have been highlighted in red in the revised manuscript for ease of reference.

**Part one-Point by point responses to editor and editorial staff comment**

1. A table should be provided in the results part that summarizes the baseline characteristics of the 161 colorectal cancer patients, such as age, sex, region of residence, clinical characteristics, and treatment types.

- **Response**: corrected accordingly. (Page 10 -14, line 200 - 218).

2. It would be more effective to restructure the interpretation of Table 3 around two thematic categories, that is, protective factors and harmful factors, instead of presenting a variable-by-variable account.

- **Response**: Confirmed and corrected accordingly and revised as Table 4. (Page 23 – 25, line 336 - 369).

3. The manuscript stated that the violation of the proportional hazards assumption was primarily driven by histological subtype, but the potential clinical or biological mechanisms underlying this phenomenon are not discussed. For instance, more aggressive subtypes may confer a higher risk early in the disease course, with the risk diminishing over time. The author should add such explanations in the discussion part to enhance the clinical interpretability of the findings.

- **Response**: We have incorporated the discussion and corrected accordingly. (Page 26 – 27, line 396 - 413).

**Reviewer 1**

1. The manuscript is very well written and informative. Nonetheless, it needs revision to make the manuscript strong and to come up with new knowledge and applicable recommendations. It is also advisable it the researcher able to show the socio-demographic characteristics of the patients hoping that these data are available in the database where he referred to relate the survival of the patients along with at least their ages as this will bring new insights.

- **Response:** Thank you for your review of our paper. We agree with this comment and have incorporated the socio-demographic characteristics and other comments accordingly. (Page 10 -14, line 200 – 218).

**Reviewer 2**

1. This manuscript addresses an important question at the intersection of oncology and survival methodology: how different survival models perform in the presence of violated proportional hazards assumptions among colorectal cancer (CRC) patients in Ethiopia. The study fills a notable methodological gap in the local literature and has potential relevance for both statisticians and clinicians. At the same time, there are several areas where the manuscript could be improved to strengthen methodological rigor, transparency, and clarity. Below I provide major and minor comments.

**Comments**

1. Clarify data source, inclusion criteria, and clinical context. The manuscript states that the dataset is from Mendeley Data and includes 161 histologically confirmed CRC cases between 2012 and 2016 in Ethiopia. Please provide more detail on:
   • How many centers contributed data and their types (e.g., tertiary hospitals, oncology centers).
   • Inclusion and exclusion criteria (e.g., primary CRC only, exclusions for missing key variables, prior cancers, etc.).
   • How loss to follow-up was defined and handled, and the proportion of patients censored due to loss vs. administrative end of follow-up.
   • A brief description of the original data collection protocol (as far as available from the Data in Brief source) would help readers understand potential selection biases.

- **Response**: Thank you for your comment. Accordingly, we have incorporated it. (Page 5 lines 104 – 106; page 6 109-115; and Page 7, 149-161).

2. Missing data and data-preprocessing strategy
   • The manuscript notes that variables with less than 10% missingness were imputed using “clinically relevant categorization or median replacement,” and that cases with incomplete survival time or unknown event status were excluded. This needs more detail:
   • For each key variable (e.g., CEA, TNM stage, metastasis, vascular invasion, treatment variables), please report the percentage of missingness and the exact method used (median imputation, categorization, listwise deletion, etc.).
   • Clarify whether imputation was performed separately by outcome status and whether any sensitivity analysis was conducted to assess the impact of imputation on model estimates.
   • Consider adding a brief table summarizing missingness and handling.

- **Response**: We agree with this and have incorporated the details (page 7, line 149 -155).

3. Model complexity versus sample size
   • You report 75 deaths among 161 patients. Given the number of prognostic factors included in the multivariable models, there is a risk of overfitting, particularly in the more complex models.
   • Please:
   • Report the exact number of parameters (including dummy variables) estimated in each final model (Cox, stratified Cox, frailty, AFT log-logistic).
   • Comment on the approximate events-per-variable ratio and justify that this is acceptable or discuss the implications for potential overfitting and wide confidence intervals.
   • Consider simplifying models (e.g., focusing on the most clinically relevant predictors) or performing sensitivity analyses to show that key findings are robust to model specification.

- **Response:** Thank you for this important comment. We agree with you and incorporated it as: The final model contained the following numbers of estimated parameters (including dummy variables for categorical predictors): Cox model 6 parameters; stratified cox model 5 parameters, frailty model 7 parameters, and AFT log-logistic model 7 parameters. Based on the observed events, the corresponding events-per-variable (EPV) ratios were approximately 12.5, 15, 10.7, and 10.7, respectively. For final study analysis the AFT model was selected. The EPV ratio of the AFT model is consistent with the conventional recommendation of at least 10 events per parameter for regression modeling, suggesting that the risk of substantial overfitting is limited. To evaluate the robustness of the findings, we performed a sensitivity analysis using simplified model. The direction and magnitude of the principal associations remained largely unchanged, supporting the robustness of the findings. (Page 26, 387-395).

4. Details of variable selection and model-building strategy
   • Variable selection is described as backward stepwise elimination guided by statistical significance and clinical relevance, but the procedure is not fully transparent.
   • Please clarify:
   • Which variables were included in the initial full model and on what basis (prior literature, clinical reasoning).
   • The threshold p-value for removal/retention during stepwise selection.
   • Whether the same set of covariates was considered in all modeling frameworks (Cox, AFT, frailty), or whether different reduced models were used.
   • Whether any checks for multicollinearity (e.g., between TNM stage, metastasis, and vascular invasion) were performed.

- **Response**: Thank you for your insightful comment. We have incorporated the description in our manuscript. (Page 7 - 8, line 149-161).

5. Handling and presentation of continuous variables (e.g., CEA, age)
   • It appears that baseline CEA may have been dichotomized (high vs. low) and that age at diagnosis may be used as a continuous or grouped variable, but this is not fully explicit.
   • Please:
   • State clearly how CEA and age were coded (continuous vs. categorized; if categorized, provide cut points and rationale).
   • Provide a brief justification for categorization, especially for CEA, given that categorization can reduce power and introduce arbitrariness.
   • Indicate whether any assessment of nonlinearity (e.g., using transformations or splines) was considered for continuous variables.

- **Response**: Thank you for comment. We agree with you.
- CEA baseline level was dichotomized as 1=elevated, and 0=normal with cutoff point elevated if >5ng/ml, else normal, from the dataset source. (Page 7, line 137-140).
- Age was treated as continuous variable. No further transformation or categorization was done. (Page 10, line 207-208).

6. Frailty model specification and interpretation
   • The frailty model is said to include a random effect for “region,” and the variance component is reported as near zero with a non-significant p-value (p = 0.93).
   • To make this result interpretable:
   • Specify the frailty distribution used (e.g., gamma or log-normal).
   • Report the number of regions and the number of patients per region, and comment on whether there was sufficient clustering to meaningfully estimate a frailty term.
   • Clarify whether this model was intended mainly as an exploratory check for unobserved heterogeneity or as a core component of your modeling strategy.

- **Response:** Thank you for point out this comment.
- We have incorporated report on the number of regions and the number of patients per region. (Page 10 -14, line 200 – 218).
- Regarding the frailty distribution, we have applied the Gaussian (normal) random effects distribution on log hazard scale. (Page 20, line 298).
- Frailty model is a type of survival model which was a candidate based on the assumption. So, if this model was satisfied the assumption of variability and being selected through the comparison, It would be selected for final study interpretation.

7. Presentation of key results and uncertainty
   • The manuscript nicely explains hazard ratios and time ratios in words, but the quantitative presentation could be improved.
   • I recommend:
   • Providing 95% confidence intervals for all key HRs and TRs in the text or tables (CEA, metastasis, vascular invasion, histology, surgery, chemotherapy).
   • For the log-logistic AFT model, explicitly linking coefficients in Table 3 to the TRs reported in the text (perhaps by adding a TR column and 95% CI).
   • Summarizing the main prognostic factors across models in a concise table that shows whether their effect direction and significance are consistent across Cox, stratified Cox, and AFT.

- **Response:** we appreciate your comment. We have incorporated accordingly. (Page 22-25 line 321-369).
- Direction and significance of main prognostic factors were consistent across the survival models. However, based on model comparison and individual models assumptions checking, we have selected AFT Log-logistic model for summarize and interpret findings of the study.

8. Interpretation of PH violations and clinical implications
   • You convincingly show that the global PH test is significant for the standard Cox model and implicate histological subtype as the main source of violation.
   • It would strengthen the paper to:
   • Show or briefly describe the Schoenfeld residual plots for histology (and any other borderline variables) to illustrate the time-varying effect.
   • Discuss in more depth what time-varying effects of histology might mean clinically (e.g., early vs. late risk differences between adenocarcinoma and squamous histology).
   • Clarify that, while the stratified Cox model resolves the PH violation by allowing separate baseline hazards by histology, it no longer estimates a direct effect for histology; this trade-off could be highlighted.

- **Response:** Thank you for this insightful comment regarding the PH assumption and its clinical interpretation. We have incorporated accordingly as: (Page 16 line 252 – 253; Page 17 line 261-267; and Page 27, line 414-319)
- Schoenfeld residual plots were added for histological subtype, confirming a time-dependent effect consistent with PH violation, while other factors met the PH assumption. Clinically, this suggests that the effect of histology on risk is stronger early in follow-up and decreases over time. To address this violation, a stratified cox model was used, allowing separate baseline hazards by histology; however this removes the ability to estimate a direct hazard ratio for histology.

9. Language and style
   • There are minor grammatical and stylistic issues throughout (e.g., “accelerate failure time” instead of “accelerated failure time,” inconsistent capitalization, and some long sentences in the Results and Discussion).
   • I suggest a careful language edit focusing on:
   • Consistent use of technical terms (Cox PH model, stratified Cox PH model, AFT model).
   • Streamlining repetitive descriptions of Kaplan–Meier curves and log-rank results (the text currently reiterates many details already apparent from the figure and table).

- **Response:** Agreed and corrected accordingly.
- Consistence use of technical words corrected accordingly.
- For the repetitive descriptions **(**Page 14, line 219 - 237**)**

10. Figure and table clarity
    • Figure 1: Ensure that axes are clearly labeled (time scale and survival probability) and that the legend is easily readable. Consider simplifying the caption to focus on the key message (progressively worse survival by stage) rather than narrating each curve in detail.
    • Table 1 and Table 2: Check alignment and formatting of columns, and ensure that abbreviations (O, E, AIC, C-index) are defined in footnotes.
    • Table 3: Adding a column for time ratios (TR) and their 95% CIs would make the clinical interpretation more straightforward.

- **Response:** Agreed and corrected accordingly. **(**Page 14 line 2229-230, Page 15 line 238 - 239, Page 22 line 334 – 335, Page 24 – 25 line 368 - 369, Page 31 line 509 - 510**)**

11. Ethics and data statement
    • The ethics section notes that ethical approval was not required because the data are fully anonymized and publicly available. It may be useful to add a brief reference to the original Data in Brief article’s ethical approvals, if described there, to reassure readers about the initial data collection.

- **Response**: Thank you for your suggestion. We have incorporated the source link. (Page 10, line 195 – 197; and page 34, line 564)
- The is publicly available in: <https://data.mendeley.com/datasets/vvzw3wkx93/3?utm_source=chatgpt.com>

12. Contextualization with Ethiopian literature
    • The Discussion appropriately cites several Ethiopian and regional studies. You might consider briefly summarizing how your key prognostic findings (CEA, metastasis, surgery, chemotherapy) compare quantitatively to hazard ratios or survival estimates reported in those prior works, to emphasize concordance or divergence.

- Response: Thank for your suggestion. We incorporate quantitative discussion. **(**Page 27, line 425-426**)**

13. Future directions
    • The recommendation to explore machine learning-based survival models is interesting. Consider specifying one or two concrete approaches (e.g., random survival forests, penalized Cox, deep learning–based survival models) and briefly stating what additional value they may provide (e.g., handling high-dimensional covariates, nonlinear effects).

- **Response:** We appreciate the reviewer’s attention to detail. We made revisions on the manuscript accordingly on the conclusion part. (Page 29 – 30, Line 470 - 481)

**Issues observed that need keen attention of the author**

14. The term region and hospital cannot go together. It is better to use the term hospital. Most importantly since the author said he applied region cluster, it would be better if it could be hospital, he should show the patients by region to see how many patients were treated in which region. Because one of the issue raised by the author is absence early detection and urgency of early surgical. These issues are related to the region and the hospital level due to far away from the capital city of the country.

- Response: Thank you for your insightful suggestion. We agreed with your comment, however the data was gathered from one hospital contains cancer patient from different regions of the country. That is why we used the region cluster.
- Description of patients by region was incorporated in the manuscript. (Page 11, line 207 - 208).

15. Since the number of patients included in the study is too small, it is difficult to generalize at national level.

- Response: Agreed, we thank the reviewer for this important comment. We acknowledge that the relatively small sample size and the single-center design limit the generalizability of our findings to the national population of colorectal cancer patients. (Page 29, line 465-468).

16. At the head of the manuscript, the name of one author only is mentioned but in the body of the manuscript, he said ‘we’. If there are other authors, their names should be mentioned otherwise the word ‘we’ should be replaced by ‘I’.

- Response: Agreed and corrected accordingly. (Page 1, line 3; and 6-10).

17. The author said in the manuscript that the patients were followed up until death. But the author said nothing how he knew these patients were followed up until their death and he relayed on the processed retrospective data only and he did not refer the patients’ charts and interviewed the health professionals where the patients were treated. Thus, it is difficult to say that the patients were followed up until death as there might be patients who were not followed up for various reasons.

- Response: Thank you for the comment. Regarding patients follow up; we have used and acknowledged the published information about the data. (Page 5, line 104 - 106).

18. The results are too general and generic. The discussion part is too shallow and was not linked with other studies as well as the results. It also lack interpretation.

- Response: Agreed and incorporated accordingly. (page 23-35, line 336-419)

19. The recommendations are too generic and does not consider other dimensions that prohibit the early detection and early surgery.

- Response: Agreed. We have revised it. (Page 30 - 31, line 485-495).

20. One result that impressed me was “the standard Cox model was not appropriate for this dataset” which was mentioned in the abstract but not indicated in the result section. This result was interesting and I was expecting the author to say something how such issues can be handled. However, the author did not say anything. I encourage the author to see this result and come up with new idea as this will be the value add of this manuscript.

- Response: Thank you for pointing out this comment. We revised and incorporated it on the result and discussion part. (Page 19 - 20, line 285 - 296)

21. References are too few. I kindly encourage the author to read more references that will enable him to analyze and interpret his data.

- Response: Accepted.
