# Supplementary material for: Survival modeling and assessing prognostic factors of colorectal cancer in Ethiopia: Addressing violation of statistical assumptions
Source: PLoS One. 2026 Oct 26;21(10):e0361233. doi: 10.1371/journal.pone.0361233 (PMC13705600; doi:10.1371/journal.pone.0361233)
Supplement: Peer review history

# Peer review: Decision Letter 0

**DOI:** 10.1371/journal.pone.0361233.r001

**Authors:** Yazhou He

12 May 2026

Dear Dr. Abraha,

Thank you for submitting your manuscript to PLOS ONE. After careful consideration, we feel that it has merit but does not fully meet PLOS ONE’s publication criteria as it currently stands. Therefore, we invite you to submit a revised version of the manuscript that addresses the points raised during the review process.

Please submit your revised manuscript by Jun 25 2026 11:59PM. If you will need more time than this to complete your revisions, please reply to this message or contact the journal office at plosone@plos.org. When you're ready to submit your revision, log on to https://www.editorialmanager.com/pone/ and select the 'Submissions Needing Revision' folder to locate your manuscript file.

- A letter that responds to each point raised by the academic editor and reviewer(s). You should upload this letter as a separate file labeled 'Response to Reviewers'.
- A marked-up copy of your manuscript that highlights changes made to the original version. You should upload this as a separate file labeled 'Revised Manuscript with Track Changes'.
- An unmarked version of your revised paper without tracked changes. You should upload this as a separate file labeled 'Manuscript'.

If you would like to make changes to your financial disclosure, please include your updated statement in your cover letter. Guidelines for resubmitting your figure files are available below the reviewer comments at the end of this letter.

If applicable, we recommend that you deposit your laboratory protocols in protocols.io to enhance the reproducibility of your results. Protocols.io assigns your protocol its own identifier (DOI) so that it can be cited independently in the future. For instructions see: https://journals.plos.org/plosone/s/submission-guidelines#loc-laboratory-protocols. Additionally, PLOS ONE offers an option for publishing peer-reviewed Lab Protocol articles, which describe protocols hosted on protocols.io. Read more information on sharing protocols at https://plos.org/protocols?utm_medium=editorial-email&utm_source=authorletters&utm_campaign=protocols.

As the corresponding author, your ORCID iD is verified in the submission system and will appear in the published article. PLOS supports the use of ORCID, and we encourage all coauthors to register for an ORCID iD and use it as well. Please encourage your coauthors to verify their ORCID iD within the submission system before final acceptance, as unverified ORCID iDs will not appear in the published article. *Only*  the individual author can complete the verification step; PLOS staff *cannot*  verify ORCID iDs on behalf of authors.

We look forward to receiving your revised manuscript.

Kind regards,

Yazhou He

Academic Editor

PLOS One

Journal Requirements:

When submitting your revision, we need you to address these additional requirements.

1. Please ensure that your manuscript meets PLOS ONE's style requirements, including those for file naming. The PLOS ONE style templates can be found at

https://journals.plos.org/plosone/s/file?id=wjVg/PLOSOne_formatting_sample_main_body.pdf and

https://journals.plos.org/plosone/s/file?id=ba62/PLOSOne_formatting_sample_title_authors_affiliations.pdf

2. Please note that PLOS One has specific guidelines on code sharing for submissions in which author-generated code underpins the findings in the manuscript. In these cases, we expect all author-generated code to be made available without restrictions upon publication of the work. Please review our guidelines at https://journals.plos.org/plosone/s/materials-and-software-sharing#loc-sharing-code and ensure that your code is shared in a way that follows best practice and facilitates reproducibility and reuse.

Additional Editor Comments:

Please address these additional concerns:

1. A table should be provided in the results part that summarizes the baseline characteristics of the 161 colorectal cancer patients, such as age, sex, region of residence, clinical characteristics, and treatment types.

2. It would be more effective to restructure the interpretation of Table 3 around two thematic categories, that is, protective factors and harmful factors, instead of presenting a variable-by-variable account.

3. The manuscript stated that the violation of the proportional hazards assumption was primarily driven by histological subtype, but the potential clinical or biological mechanisms underlying this phenomenon are not discussed. For instance, more aggressive subtypes may confer a higher risk early in the disease course, with the risk diminishing over time. The author should add such explanations in the discussion part to enhance the clinical interpretability of the findings.

[Note: HTML markup is below. Please do not edit.]

Reviewers' comments:

Reviewer's Responses to Questions

**Comments to the Author**

1. Is the manuscript technically sound, and do the data support the conclusions?

Reviewer #1: Yes

Reviewer #2: Partly

**********

2. Has the statistical analysis been performed appropriately and rigorously?

Reviewer #1: Yes

Reviewer #2: Yes

**********

3. Have the authors made all data underlying the findings in their manuscript fully available?

The PLOS Data policy

Reviewer #1: No

Reviewer #2: Yes

**********

4. Is the manuscript presented in an intelligible fashion and written in standard English?

Reviewer #1: Yes

Reviewer #2: Yes

**********

Reviewer #1: The manuscript is very well written and informative. Nonetheless, it needs revision to make the manuscript strong and to come up with new knowledge and applicable recommendations. It is also advisable it the researcher able to show the socio-demographic characteristics of the patients hoping that these data are available in the database where he referred to relate the survival of the patients along with at least their ages as this will bring new insights.

Reviewer #2: This manuscript addresses an important question at the intersection of oncology and survival methodology: how different survival models perform in the presence of violated proportional hazards assumptions among colorectal cancer (CRC) patients in Ethiopia. The study fills a notable methodological gap in the local literature and has potential relevance for both statisticians and clinicians. At the same time, there are several areas where the manuscript could be improved to strengthen methodological rigor, transparency, and clarity.

Below I provide major and minor comments.

________________________________________

Major comments

1. Clarify data source, inclusion criteria, and clinical context

• The manuscript states that the dataset is from Mendeley Data and includes 161 histologically confirmed CRC cases between 2012 and 2016 in Ethiopia. Please provide more detail on:

• How many centers contributed data and their types (e.g., tertiary hospitals, oncology centers).

• Inclusion and exclusion criteria (e.g., primary CRC only, exclusions for missing key variables, prior cancers, etc.).

• How loss to follow-up was defined and handled, and the proportion of patients censored due to loss vs. administrative end of follow-up.

• A brief description of the original data collection protocol (as far as available from the Data in Brief source) would help readers understand potential selection biases.

2. Missing data and data-preprocessing strategy

• The manuscript notes that variables with less than 10% missingness were imputed using “clinically relevant categorization or median replacement,” and that cases with incomplete survival time or unknown event status were excluded. This needs more detail:

• For each key variable (e.g., CEA, TNM stage, metastasis, vascular invasion, treatment variables), please report the percentage of missingness and the exact method used (median imputation, categorization, listwise deletion, etc.).

• Clarify whether imputation was performed separately by outcome status and whether any sensitivity analysis was conducted to assess the impact of imputation on model estimates.

• Consider adding a brief table summarizing missingness and handling.

3. Model complexity versus sample size

• You report 75 deaths among 161 patients. Given the number of prognostic factors included in the multivariable models, there is a risk of overfitting, particularly in the more complex models.

• Please:

• Report the exact number of parameters (including dummy variables) estimated in each final model (Cox, stratified Cox, frailty, AFT log-logistic).

• Comment on the approximate events-per-variable ratio and justify that this is acceptable or discuss the implications for potential overfitting and wide confidence intervals.

• Consider simplifying models (e.g., focusing on the most clinically relevant predictors) or performing sensitivity analyses to show that key findings are robust to model specification.

4. Details of variable selection and model-building strategy

• Variable selection is described as backward stepwise elimination guided by statistical significance and clinical relevance, but the procedure is not fully transparent.

• Please clarify:

• Which variables were included in the initial full model and on what basis (prior literature, clinical reasoning).

• The threshold p-value for removal/retention during stepwise selection.

• Whether the same set of covariates was considered in all modeling frameworks (Cox, AFT, frailty), or whether different reduced models were used.

• Whether any checks for multicollinearity (e.g., between TNM stage, metastasis, and vascular invasion) were performed.

5. Handling and presentation of continuous variables (e.g., CEA, age)

• It appears that baseline CEA may have been dichotomized (high vs. low) and that age at diagnosis may be used as a continuous or grouped variable, but this is not fully explicit.

• Please:

• State clearly how CEA and age were coded (continuous vs. categorized; if categorized, provide cut points and rationale).

• Provide a brief justification for categorization, especially for CEA, given that categorization can reduce power and introduce arbitrariness.

• Indicate whether any assessment of nonlinearity (e.g., using transformations or splines) was considered for continuous variables.

6. Frailty model specification and interpretation

• The frailty model is said to include a random effect for “region,” and the variance component is reported as near zero with a non-significant p-value (p = 0.93).

• To make this result interpretable:

• Specify the frailty distribution used (e.g., gamma or log-normal).

• Report the number of regions and the number of patients per region, and comment on whether there was sufficient clustering to meaningfully estimate a frailty term.

• Clarify whether this model was intended mainly as an exploratory check for unobserved heterogeneity or as a core component of your modeling strategy.

7. Presentation of key results and uncertainty

• The manuscript nicely explains hazard ratios and time ratios in words, but the quantitative presentation could be improved.

• I recommend:

• Providing 95% confidence intervals for all key HRs and TRs in the text or tables (CEA, metastasis, vascular invasion, histology, surgery, chemotherapy).

• For the log-logistic AFT model, explicitly linking coefficients in Table 3 to the TRs reported in the text (perhaps by adding a TR column and 95% CI).

• Summarizing the main prognostic factors across models in a concise table that shows whether their effect direction and significance are consistent across Cox, stratified Cox, and AFT.

8. Interpretation of PH violations and clinical implications

• You convincingly show that the global PH test is significant for the standard Cox model and implicate histological subtype as the main source of violation.

• It would strengthen the paper to:

• Show or briefly describe the Schoenfeld residual plots for histology (and any other borderline variables) to illustrate the time-varying effect.

• Discuss in more depth what time-varying effects of histology might mean clinically (e.g., early vs. late risk differences between adenocarcinoma and squamous histology).

• Clarify that, while the stratified Cox model resolves the PH violation by allowing separate baseline hazards by histology, it no longer estimates a direct effect for histology; this trade-off could be highlighted.

________________________________________

Minor comments

1. Language and style

• There are minor grammatical and stylistic issues throughout (e.g., “accelerate failure time” instead of “accelerated failure time,” inconsistent capitalization, and some long sentences in the Results and Discussion).

• I suggest a careful language edit focusing on:

• Consistent use of technical terms (Cox PH model, stratified Cox PH model, AFT model).

• Streamlining repetitive descriptions of Kaplan–Meier curves and log-rank results (the text currently reiterates many details already apparent from the figure and table).

2. Figure and table clarity

• Figure 1: Ensure that axes are clearly labeled (time scale and survival probability) and that the legend is easily readable. Consider simplifying the caption to focus on the key message (progressively worse survival by stage) rather than narrating each curve in detail.

• Table 1 and Table 2: Check alignment and formatting of columns, and ensure that abbreviations (O, E, AIC, C-index) are defined in footnotes.

• Table 3: Adding a column for time ratios (TR) and their 95% CIs would make the clinical interpretation more straightforward.

3. Ethics and data statement

• The ethics section notes that ethical approval was not required because the data are fully anonymized and publicly available. It may be useful to add a brief reference to the original Data in Brief article’s ethical approvals, if described there, to reassure readers about the initial data collection.

4. Contextualization with Ethiopian literature

• The Discussion appropriately cites several Ethiopian and regional studies. You might consider briefly summarizing how your key prognostic findings (CEA, metastasis, surgery, chemotherapy) compare quantitatively to hazard ratios or survival estimates reported in those prior works, to emphasize concordance or divergence.

5. Future directions

• The recommendation to explore machine learning-based survival models is interesting. Consider specifying one or two concrete approaches (e.g., random survival forests, penalized Cox, deep learning–based survival models) and briefly stating what additional value they may provide (e.g., handling high-dimensional covariates, nonlinear effects).

**********

what does this mean?). If published, this will include your full peer review and any attached files.

If you choose “no”, your identity will remain anonymous but your review may still be made public.

**Do you want your identity to be public for this peer review?**  For information about this choice, including consent withdrawal, please see our Privacy Policy

Reviewer #1: **Yes:**  Tamiru Demeke

Reviewer #2: **Yes:**  Faisel Abdi Hassen

**********

[NOTE: If reviewer comments were submitted as an attachment file, they will be attached to this email and accessible via the submission site. Please log into your account, locate the manuscript record, and check for the action link "View Attachments". If this link does not appear, there are no attachment files.]

To ensure your figures meet our technical requirements, please review our figure guidelines: https://journals.plos.org/plosone/s/figures

You may also use PLOS’s free figure tool, NAAS, to help you prepare publication quality figures: https://journals.plos.org/plosone/s/figures#loc-tools-for-figure-preparation.

NAAS will assess whether your figures meet our technical requirements by comparing each figure against our figure specifications.

Attachment Submitted filename: renamed_82689.docx

# Author comment: Author response to Decision Letter 1

**DOI:** 10.1371/journal.pone.0361233.r002

17 Jul 2026

We sincerely thank you for your thorough and constructive feedback. We have carefully addressed all the comments and concerns raised, ensuring a more cautious interpretation of the findings and improving the overall clarity of the manuscript. In addition, we have refined the language, corrected grammatical and spelling errors, reference formatting, and citation consistency, and strengthened the presentation of the content.

Attachment Submitted filename: Response to Reviewers.docx

# Peer review: Decision Letter 1

**DOI:** 10.1371/journal.pone.0361233.r003

**Authors:** Yazhou He

8 Oct 2026

Survival Modeling and Assessing Prognostic Factors of Colorectal Cancer in Ethiopia: Addressing Violation of Statistical Assumptions

PONE-D-26-16119R1

Dear Dr. Abraha,

We’re pleased to inform you that your manuscript has been judged scientifically suitable for publication and will be formally accepted for publication once it meets all outstanding technical requirements.

Within one week, you’ll receive an e-mail detailing the required amendments. When these have been addressed, you’ll receive a formal acceptance letter and your manuscript will be scheduled for publication.

An invoice will be generated when your article is formally accepted. Please note, if your institution has a publishing partnership with PLOS and your article meets the relevant criteria, all or part of your publication costs will be covered. Please make sure your user information is up-to-date by logging into Editorial Manager at Editorial Manager® and clicking the ‘Update My Information' link at the top of the page. For questions related to billing, please contact billing support.

If your institution or institutions have a press office, please notify them about your upcoming paper to help maximize its impact. If they’ll be preparing press materials, please inform our press team as soon as possible -- no later than 48 hours after receiving the formal acceptance. Your manuscript will remain under strict press embargo until 2 pm Eastern Time on the date of publication. For more information, please contact onepress@plos.org.

Kind regards,

Yazhou He

Academic Editor

PLOS One

Additional Editor Comments (optional):

Reviewers' comments:

Reviewer's Responses to Questions

**Comments to the Author**

Reviewer #1: All comments have been addressed

Reviewer #2: All comments have been addressed

**********

2. Is the manuscript technically sound, and do the data support the conclusions?

Reviewer #1: Yes

Reviewer #2: Yes

**********

3. Has the statistical analysis been performed appropriately and rigorously?

Reviewer #1: Yes

Reviewer #2: Yes

**********

4. Have the authors made all data underlying the findings in their manuscript fully available?

The PLOS Data policy

Reviewer #1: Yes

Reviewer #2: Yes

**********

5. Is the manuscript presented in an intelligible fashion and written in standard English?

Reviewer #1: Yes

Reviewer #2: Yes

**********

Reviewer #1: The authors addressed all my comments and the manuscript is now very well improved. Therefore, I recommend the manuscript to be published

# Editor report: Acceptance letter

**DOI:** 10.1371/journal.pone.0361233.r004

**Authors:** Yazhou He

PONE-D-26-16119R1

PLOS One

Dear Dr. Abraha,

I'm pleased to inform you that your manuscript has been deemed suitable for publication in PLOS One. Congratulations! Your manuscript is now being handed over to our production team.

At this stage, our production department will prepare your paper for publication. This includes ensuring the following:

* All references, tables, and figures are properly cited

* All relevant supporting information is included in the manuscript submission,

* There are no issues that prevent the paper from being properly typeset

You will receive further instructions from the production team, including instructions on how to review your proof when it is ready. Please keep in mind that we are working through a large volume of accepted articles, so please give us a few days to review your paper and let you know the next and final steps.

Lastly, if your institution or institutions have a press office, please let them know about your upcoming paper now to help maximize its impact. If they'll be preparing press materials, please inform our press team within the next 48 hours. Your manuscript will remain under strict press embargo until 2 pm Eastern Time on the date of publication. For more information, please contact onepress@plos.org.

You will receive an invoice from PLOS for your publication fee after your manuscript has reached the completed accept phase. If you receive an email requesting payment before acceptance or for any other service, this may be a phishing scheme. Learn how to identify phishing emails and protect your accounts at https://explore.plos.org/phishing.

If we can help with anything else, please email us at customercare@plos.org.

Thank you for submitting your work to PLOS One and supporting open access.

Kind regards,

PLOS One Editorial Office Staff

on behalf of

Dr. Yazhou He

Academic Editor

PLOS One
